# *Wolbachia* Induces Density-Dependent Inhibition to Dengue Virus in Mosquito Cells

**DOI:** 10.1371/journal.pntd.0001754

**Published:** 2012-07-24

**Authors:** Peng Lu, Guowu Bian, Xiaoling Pan, Zhiyong Xi

**Affiliations:** 1 Department of Microbiology and Molecular Genetics, Michigan State University, East Lansing, Michigan, United States of America; 2 Department of Entomology, Michigan State University, East Lansing, Michigan, United States of America; Monash University, Australia

## Abstract

*Wolbachia* is a maternal transmitted endosymbiotic bacterium that is estimated to infect up to 65% of insect species. The ability of *Wolbachia* to both induce viral interference and spread into mosquito vector population makes it possible to develop *Wolbachia* as a biological control agent for dengue control. While *Wolbachia* induces resistance to dengue virus in the transinfected *Aedes aegypti* mosquitoes, a similar effect was not observed in *Aedes albopictus*, which naturally carries *Wolbachia* infection but still serves as a dengue vector. In order to understand the mechanism of this lack of *Wolbachia*-mediated viral interference, we used both *Ae. albopictus* cell line (Aa23) and mosquitoes to characterize the impact of *Wolbachia* on dengue infection. A serial of sub-lethal doses of antibiotic treatment was used to partially remove *Wolbachia* in Aa23 cells and generate cell cultures with *Wolbachia* at different densities. We show that there is a strong negative linear correlation between the genome copy of *Wolbachia* and dengue virus with a dengue infection completely removed when *Wolbacha* density reaches a certain level. We then compared *Wolbachia* density between transinfected *Ae. aegypti* and naturally infected *Ae. albopictus*. The results show that *Wolbachia* density in midgut, fatbody and salivary gland of *Ae. albopictus* is 80-, 18-, and 24-fold less than that of *Ae. aegypti*, respectively. We provide evidence that *Wolbachia* density in somatic tissues of *Ae. albopictus* is too low to induce resistance to dengue virus. Our results will aid in understanding the mechanism of *Wolbachia*-mediated pathogen interference and developing novel methods to block disease transmission by mosquitoes carrying native *Wolbachia* infections.

## Author Summary

Transmitted by mosquitoes, dengue virus causes the most important arbovirus disease in humans. Increasing problems in insecticide resistance and the lack of drugs and vaccines make it urgent to develop novel strategies for dengue control. *Wolbachia* is a maternally transmitted Gram-negative endosymbiotic bacterium that infects approximately 28% of mosquito species. It can not only spread within mosquito populations through its unique ability to manipulate mosquito reproduction but can also induce resistance to dengue virus in mosquito vectors. This leads to a genetic control strategy in which mosquito hosts are made to be inhospitable to dengue virus through population replacement. However, it is challenged by the fact that *Ae. albopictus* naturally carries *Wolbachia* infections but still services as a dengue vector. In this study we show the native *Wolbachia* induces a resistance to dengue virus in *Wolbachia* density-dependent manner in *Ae. albopictus*. With a decrease in *Wolbachia* density within the host cells, dengue infection increases dramatically. We provide evidence that a very low *Wolbachia* density in mosquito tissues where dengue virus will reside and travel could contribute to absence of *Wolbachia*-mediated resistance to dengue virus in *Ae. albopictus*.

## Introduction

Dengue fever and dengue hemorrhagic fever are emerging globally as a major public health problem in more than 100 countries. 2.5 to 3.0 billion people, or two fifths of the world's populations, are currently living in dengue-endemic areas. Each year there are an estimated 100 million new dengue cases and 22,000 deaths [1]. The number of cases reported to WHO has increased nearly nine times in the past 4 decades, and it continues to expand into temperate climates. Its fatal form, dengue hemorrhagic fever, has expended from Southeast Asia to 28 countries in the Western Hemisphere. Dengue virus (DENV) is transmitted to humans by the mosquitoes *Aedes aegypti* and *Aedes albopictus*. Currently no drug therapy or vaccines are available for dengue fever. Vector control is the primary intervention tool, but this barrier is weakened by increased pesticide resistance. This situation justifies seeking alternative and innovative approaches for the development of new prevention options. *Wolbachia*-based dengue control strategy is showing great potential because it could provide a solution that will be more sustainable, economical and environmental friendly than the other methods [2].

*Wolbachia* spp. are intracellular alpha-proteobacteria closely related to *Rickettsia*. Maternally inherited infections with *Wolbachia* occur in more than 65% of all the insect species and approximately 28% of the surveyed mosquito species [3], [4]. Through the cytoplasmic incompatibility (CI) mechanism, *Wolbachia* can induce early embryo death when a *Wolbachia*-infected male mates with an uninfected female [5]. Since infected females can successfully mate with uninfected males, *Wolbachia* can spread quickly in a population. This has been observed to occur naturally in *Drosophila simulans* [6], and demonstrated in *Ae. aegypti* through both laboratory cage studies and a recent field trial [7], [8].

Another important feature of *Wolbachia* is its ability to induce resistance to a variety of pathogens, including DENV, in its insect hosts [9], [10], [11], [12], [13], [14], [15], [16]. In the transinfected *Ae. aegypti*, all the three different types of *Wolbachia*, *w*AlbB, *w*MelPop-CLA, and *w*Mel, show a significant inhibition to replication and dissemination of DENV, resulting in either complete or partial block of viral transmission [9], [12], [13]. Recent studies further show that *Wolbachia* induces production of reactive-oxygen species (ROS) which then activate Toll-pathway to induce expression of antiviral effectors [17]. In the *Drosophila* host, native *Wolbachia* can also confer resistance to DENV and the other pathogens [15]. This resistance appears to be induced by the non-immune related mechanisms because the tested immune genes do not show differential expression in response to *Wolbachia* [18], [19]. Inhibition of dengue virus replication was also observed in cell lines, and the extent of inhibition is related to bacterial density [20].

*Ae. albopictus* is generally considered as the secondary vector of DENV. This mosquito species naturally carries two types of *Wolbachia*, *w*AlbA and *w*AlbB, which distribute throughout both germ line and somatic tissues in mosquitoes [21]. However, *Wolbachia*-mediated viral interference was not observed in *Ae. albopictus* [12], consistent with the fact that it is a competent vector for at least 22 arboviruses [22]. The recent study shows a transinfected *Ae. albopictus* line that carries *w*Mel is resistant to DENV [23], which excludes the possibility that *Ae. albopictus* lacks the genetic background for *Wolbachia* to induce viral interference. Thus, although both *w*AlbB and *Ae. albopictus* own the machinery to induce viral interference, the resistance to DENV does not occur for unknown reasons.

In this work, we used an Aa23 cell line, initially established from eggs of *Ae. albopictus* [24], to study the mechanism underlying the lack of *Wolbachia*-mediated resistance to DENV. We confirmed that *w*AlbB induces a strong resistance to DENV in Aa23 cell line. Moreover, the levels of resistance strongly correlate with density of *Wolbachia* in Aa23 cells. Further comparison of genome copy of *w*AlbB in both somatic and germ line tissues between dengue resistant transinfected *Ae. aegypti* and susceptible *Ae. albopictus* suggests that *Wolbachia* density is too low to induce resistance to DENV in *Ae. albopictus*.

## Methods

### Mosquito rearing and cell culture maintenance

Houston (HOU) and HT1 strain of *Ae. albopictus* and WB1 strain of *Ae. aegypti* were maintained at 27°C and RH 85% with a 12-hr light/dark cycle. *Ae. albopictus* Aa23, Aa23T cell lines (derived from Aa23 cells through tetracycline treatment) and *Ae. aegypti* Aag2 cell line were cultured as previously described [25], [26]. Those cells were maintained in Schneider's *Drosophila* Medium (Invitrogen) supplemented with 10% (v/v) heat-inactivated fetal bovine serum (FBS).

### Introduction of *Wolbachia* into Aag2 cells

*w*-Aag2 cell line was generated by introducing *Wolbachia* from Aa23 cells into Aag2 cell line using shell vial technique as previously described [25] with a slight modification. In brief, two 75-cm^2^ flasks of confluent Aa23 cells were shaken, centrifuged at 1,000× g for 10 min, re-suspended in 1.5 ml Schneider's *Drosophila* Medium in a 50 ml conical tube, and lysed by votexing with 3-mm-diameter glass beads. The lysate was centrifuged at 2,500× g for 10 min, and the supernatant was filtered through a 5 µm syringe filter (Millipore). 500 µl of the filtrate was overlaid on Aag2 cells grown in 12-well plate to 80% confluence. The plate was centrifuged at 2,000× g at 15°C for 1 h and then incubated at 26°C overnight. After that, the cells were transferred to a 25 ml cell flask with fresh medium. *Wolbachia* was stably maintained in *w*-Aag2 for more than 17 passages before being used for dengue infection.

### Density-dependent assay

To generate Aa23 cells with different *Wolbachia* densities, the cell culture was treated with rifampicin to make a final concentration at 5 µg/ml, 0.5 µg/ml and 0.05 µg/ml in 24-well plates. For each dose, cells were treated for four time periods: 5 h, 10 h, 40 h and 70 h. After treatment, the old medium was removed and cells were washed with fresh medium for one time. After adding fresh medium, cells were grown for another three days. After that, cells were passaged to another 24 well-plate with new medium. Two days after the passage, cells were infected with DENV-2 at multiplicity of infection (MOI) of 0.1. At Day 5 post infection, cells were collected to measure the genome copy of *Wolbachia* and DENV-2. To study *Wolbachia*-density dependent expression of Defensin D (DEFD), cells were grown for 7 days in fresh medium after rifampicin treatment. Then, cells were collected to measure both the genome copy of *Wolbachia* and the expression of DEFD [27].

### DENV-2 Infections in Mosquitoes

The New Guinea C strain of DENV serotype 2 (DENV-2) was propagated in C6/36 cells as previously described [28]. Virus was harvested 7 days post infection by collection of supernatants and centrifugation at 3,000× g. The virus suspension was mixed in 1∶1 with commercial ox defibrinated blood (Colorado Serum Company). The blood meal was warmed at 37°C in water bath for 30 min and then used to feed 7-day-old mosquitoes as described previously [29].

### RNA Extraction, cDNA Synthesis and Quantitative Reverse Transcription Polymerase Chain Reaction (qRT-PCR)

The total RNA or viral genomic RNA were extracted from either cell lines or mosquitoes tissues by RNeasy Mini Kit (QIAGEN Sciences, Germantown, MD, USA) and then the cDNA were transcript using QuantiTect reverse Transcription Kit (QIAGEN Sciences, Germantown, MD, USA). Real-time PCR was performed using the QuantiTect SYBR Green PCR Kit (QIAGEN Sciences, Germantown, MD, USA) and ABI Prism 7900HT Sequence Detection System (Applied Biosystems, Foster City, CA, USA). DENV-2 genomic RNA was measured by qRT-PCR using primers directed to NS5 gene [30]. The dengue copy was normalized with host actin gene [31]. A standard curve was generated for each of NS5 and actin by analyzing 10^1^ to 10^8^ copies/reaction of the two plasmids containing each individual fragment [31]. DENV-2 in mosquito head was diagnosed by RT-PCR with the same NS5 primers. DEFD was amplified with the primers: For (5′-GTCTGTTGCCAACTCTCTTT -3′) and Rev (5′- CACAAGCACTGTCACCAAC -3′).

### *Wolbachia* Quantitative PCR (q-PCR)

qPCR was performed to measure the *Wolbachia* density in the cell lines and mosquitoes [31]. Genomic DNA was extracted from either Aa23 cells (two to six biological replicates) or mosquito tissues (ten biological replicates). The primers specifically directed to *Wolbachia* surface protein (wsp) of *w*AlbA and *w*AlbB were used in PCR to measure the *Wolbachia* genome copy, which was normalized with the host actin for Aa23 cells [31] or ribosomal protein S6 (RPS6) gene for both *Ae. albopictus* and *Ae. aegypti* mosquitoes. RPS6 was amplified with the primers: For (5′-GAAGTTGAACGTATCGTTTC-3′) and Rev (5′-GAGATGGTCAGCGGTGATTT-3′). A standard curve was generated for each of *w*AlbA, *w*AlbB and actin by analyzing 10^1^ to 10^8^ copies/reaction of the pQuantAlb plasmid [31]. Another plasmid containing the RPS6 fragment was cloned to generate another standard curve for RPS6.

### Susceptibility of the host cells to DENV infection

The susceptibility of Aa23, Aa23T, *w*-Aag2 and Aag2 cell lines for DENV-2 was measured by plaque assay with a slight modification [29]. DENV-2 virus stock (2×10^7^ PFU/ml; determined previously in C6/36 cells) was serially diluted in ten-fold increments (from 10^1^ to 10^6^) using cell medium, and then inoculated into each of the four mosquito cells in 24-well plates. After incubation at 26°C for 5 days, the plates were assayed for plaque formation by peroxidase immunostaining, using mouse hyperimmune ascitic fluid and a goat anti-mouse HRP conjugate as the primary and secondary antibody. At least five biological replicates were used for each treatment.

### Immunofluorescence

The viral antigen and *Wolbachia* wsp protein in Aa23 cells were detected simultaneously using an indirect immunofluorescence assay (IFA). Confluent rifampicin -treated Aa23 cells were grown on chamber slides (Thermo), and then were infected with DENV-2 at 0.1 MOI. After incubation for 2 days, cells were fixed for 30 min at 4°C in 4% (w/v) paraformaldehyde in PBS, containing 0.5% (v/v) Triton X-100. Then, samples were incubated in PBS and 10% (v/v) goat serum at room temperature for 1 h. Subsequently, the slides were incubated with a mouse anti-dengue complex monoclonal antibody at a 1∶300 dilution and a rabbit anti-wsp polyclonal antibody (GenScript) at a 1∶500 dilution in PBS at room temperature for 1 h. After wash, the slides were incubated with Alexa-488 anti-mouse and Alexa-594 anti-rabbit antibodies (Molecular Probes, Invitrogen) at a 1∶1000 dilution in PBS at room temperature for 1 h. To stain cell nucleus, the samples were incubated with DAPI for 10 min. Immunostaining was examined under with an Olymupus Fluoview 1000 confocal microscope.

## Results

### The *Wolbachia w*AlbB induce strong resistance to DENV-2 in *Ae. albopictus* Aa23 cells

**Figure 1:**
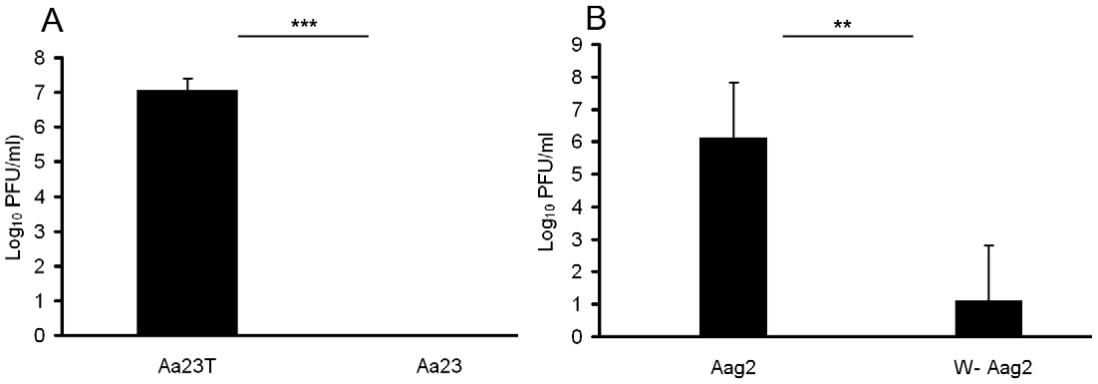
*w*AlbB induces a strong resistance to DENV-2 in mosquito cells. *w*AlbB is a native infection in *Ae. albopictus* Aa23 cells (**A**) while Aa23T cells were initially generated by tetracycline treatment of Aa23 cell to remove *Wolbachia* infection. There is no *Wolbachia* in *Ae. aegypti* Aag2 cells (**B**). *w*-Aag2 was generated from Aag2 cells by introducing *w*AlbB from Aa23 using a shell-vial technique. Five days after inoculated with DENV-2, the cells were tested for dengue infection by plaque assay. Error bars are standard errors of the mean of at least three biological replicates. **, P<0.01; ***, P<0.001in Student's t-Test.

We previously reported that the *Wolbachia w*AlbB does not inhibit dissemination of DENV-2 to the mosquito head in the naturally infected *Ae. albopictus* [12]. This could indicate that *w*AlbB does not induce resistance to DENV in the host genetic background of *Ae. albopictus*. To test that possibility, we compared susceptibility for DENV-2 between *w*AlbB-infected Aa23 cell line and its aposymbiotic Aa23T. Seven day after viral infection, the viral titer in Aa23T reached to 1.1×10^7^ PFU/ml while no virus particle was detected in Aa23 cell line (Fig. 1A). A parallel assay was conducted to measure the *Wolbachia*-mediated viral interference in *Ae. aegypti* cell line Aag2. Like *Ae. aegypti* mosquito, Aag2 cell line is uninfected by *Wolbachia*. We introduced *w*AlbB from Aa23 into Aag2 through a shell-vial technique, generating a *Wolbachia*-infected cell line *w*-Aag2. As expected, viral titer in the Aag2 cell line (1.3×10^6^ PFU/ml) is significantly higher than in the *w*-Aag2 cell line (12.5 PFU/ml; P<0.01, Student's t-Test; Fig. 1B). These results confirm that *w*AlbB can induce resistance to DENV-2 even in *Ae. albopictus* host background.

### Generation of Aa23 cell lines infected with *Wolbachia* at different densities

**Figure 2:**
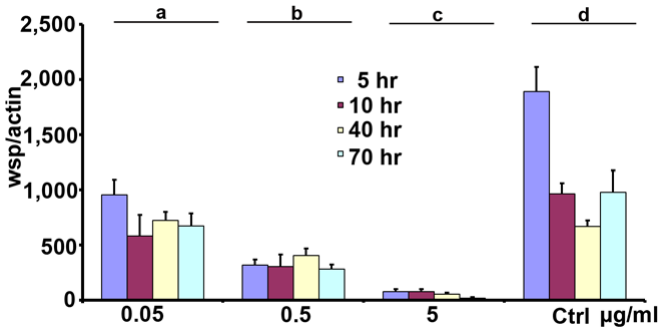
Generation of Aa23 cells with different *Wolbachia* density. Cells were treated using sub-lethal doses of rifampicin for a different time periods. Three dosages (0.05 µg/ml, 0.5 µg/ml and 5 µg/ml) and four time periods (4 h, 10 h, 40 h and 70 h) were used. The genome copies of wsp were measure by q-PCR, normalized by host gene actin. Error bars are standard errors of the mean of at least three biological replicates. Statistical significance is represented by letters above each column, with different letters signifying distinct statistical groups. Student's t test: a vs. b, P<0.001; b vs. c, P<0.001; d vs. a, P<0.05.

To study how the density of *w*AlbB can influence its induced resistance to DENV in Aa23 cell line, we used sub-lethal doses of rifampicin to partially remove *Wolbachia* and generated cell cultures with *Wolbachia* at different densities. Three dosages (0.05 µg/ml, 0.5 µg/ml and 5 µg/ml) with four treatment times (4 h, 10 h, 40 h and 70 h), resulting in a total of 12 treatments, were designed to make *Wolbachia* density cover a broad range. A mock treatment (control) was included to treat cells with only methanol solvent for the above four time periods. As a result (Fig. 2), we generated a serial of cell cultures with different *Wolbachia* densities, with a highest at 953.2 wsp/actin (from 0.05 µg/ml and 4 h) and a lowest at 18.0 wsp/actin (from 5 µg/ml and 70 h). *Wolbachia* density significantly decreased with an increase in treatment dose. Within each dosage, no significant difference in *Wolbachia* density is observed among four treatment times. This indicates a treatment for 4 h can effectively remove *Wolbachia* and a further increase in treatment time will not significantly enhance the inhibitory effect of rifampicin on *Wolbachia*. For example, a treatment at dosage of 5 µg/ml for 4 h can dramatically reduce *Wolbachia* to a very low level (75.2 wsp/actin), compared to the mock treatment (1,888.3 wsp/actin). Further increasing treatment time at this dose will not significantly change the *Wolbachia* density, resulting in 74.1 wsp/actin (10 h), 52.4 wsp/actin (40 h), and 18.0 wsp/actin (70 h, Fig. 2).

### The *Wolbachia w*AlbB induces resistance to DENV in a density-dependent manner

**Figure 3:**
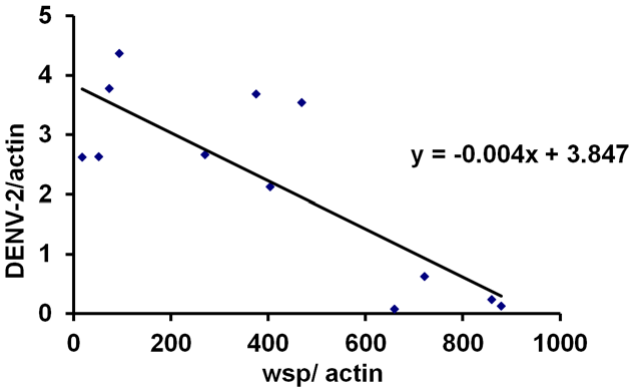
*Wolbachia* induces density-dependent inhibition to DENV-2 in Aa23 cell lines. Cell cultures derived from twelve different treatments in Fig. 2 were used in the assay. Five days after these cells were infected with DENV-2, viral genome copies were measured by qRT-PCR. Actin was used as a host gene to normalize the data. There is a negative linear correlation between *Wolbachia* density and DENV copy. Each point is the mean of at least three biological replicates.

To study the relationship between *Wolbachia* density and dengue infection level, cell cultures derived from the above 12 treatments were inoculated with DENV-2. Five days after infection, we measured genome copies of DENV-2 in the cells. *Wolbachia* density in the cells and its corresponding viral infection level were recorded in parallel to examine their interactions. We observed that genome copy of DENV-2 in cells increased with a decrease in *Wolbachia* density. Moreover, there is a negative correlation between the genome copy of *Wolbachia* and DENV-2 (r = −0.82; P<0.001). The level of DENV-2 (y) can be predicted by the genome copy of *Wolbachia* (x) via a model: y = −0.004x+3.847 (Fig. 3). These results confirm that *Wolbachia* induces a density-dependent viral inhibition in Aa23 cells.

### *Wolbachia* density in somatic tissues of *Ae. albopictus* is too low to induce viral interference

**Figure 4:**
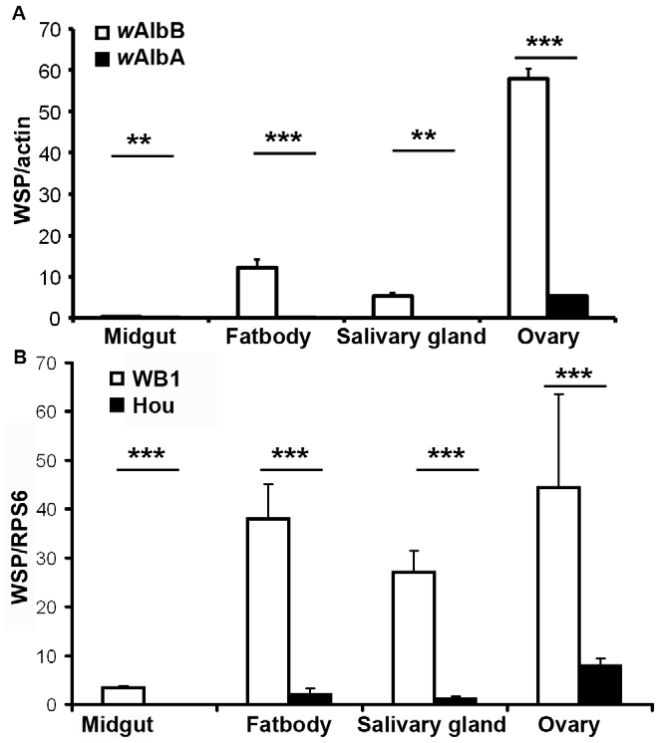
*Wolbachia* density in somatic tissues of *Ae. albopictus* is too low to induce resistance to DENV. (A). The density of *w*AlbA is significantly lower than *w*AlbB in midgut, fatbody, salivary gland and ovary of *Ae. albopictus*. The copy number of the *Wolbachia* wsp was normalized by *Ae. albopictus* actin; (B). *Wolbachia* density in somatic tissues is significantly lower in the *Ae. albopictus* HOU strain than in the transinfected *Ae. aegypti* WB1 strain. The copy number of the *Wolbachia* wsp was normalized by one conserved RPS6 in both *Ae. albopictus* and *Ae. aegypti*. In all the assays, midguts, salivary glands, fatbodies and ovaries of 7-day-old non blood fed females were dissected and used for extraction of total genomic DNA. ***, P<0.001; **, P<0.01 in Student's t-Test. Error bars are standard errors of the mean of ten biological replicates.

Within mosquito vectors, DENV needs replicate and pass through midguts and salivary glands before transmission occurs. *Wolbachia*-mediated density-dependent viral inhibition in mosquito cells leads to a hypothesis that the *Wolbachia* density in the somatic tissues of *Ae. albopictus* is too low to induce viral interference in this mosquito species. Thus, we measured *Wolbachia* density in both somatic tissues (midgut, salivary gland, fatbody) and germ line tissue (ovary) of the HOU strain of *Ae. albopictus* (carrying *w*AlbA and *w*AlbB). As shown in Fig. 4A, *w*AlbB is significantly higher than *w*AlbA in all the four tissues. While none of *w*AlbA could be detected in midgut, salivary gland and fatbody, only 5.4 wsp/actin of wAlbA presents in ovary. There are 0.3, 5.3, and 12.3 wsp/actin of *w*AlbB in midgut, salivary gland and fatbody, respectively, compared to 57.9 wsp/actin of *w*AlbB in ovary.

To better compare *Wolbachia* density between the HOU strain of *Ae. albopictus* and the transinfected *Ae. aegypti* WB1 strain (carrying *w*AlbB), we designed one set of primers that can amplify a conserved region of Ribosomal protein S6 (RPS6) in both mosquito species, which was used to normalize the *Wolbachia* copy in qRT-PCR. We observed that *w*AlbB density is significantly lower in the midgut than in the ovary and salivary gland for both mosquito species (P<0.05, Student's t-Test). However, *w*AlbB density in fatbody is similar to that in salivary gland. Importantly, a significant lower density of *w*AlbB presents in midgut, fatbody and salivary gland (P<0.001, Student's t-Test) of HOU mosquitoes as compared with that of WB1 mosquitoes. Specifically, the *Wolbachia* genome copies in midgut, fatbody and salivary gland of HOU are 80-, 18-, and 24-fold less than that of WB1, respectively (Fig. 4B). In ovary, *w*AlbB density is also 5.6-fold lower in HOU than in WB1 (P<0.001, Mann-Whiteney U test).

To confirm a lack of *Wolbachia*-mediated viral interference in *Ae. albopictus*, we compared mosquito susceptibility for DENV-2 at a serial diluted viral titers between the HOU and its aposymbiotic strain HT1 (initially derived from Houston strain through tetracycline treatment). We have previously observed that *Wolbachia* suppresses viral dissemination in the HOU mosquitoes after mosquitoes took infectious blood meal at a viral titer of 10^7^ PFU/ml [12]. However, it is possible that *Wolbachia* induces only a weak resistance to DENV in *Ae. albopictus* which could be overcome when a very high viral titer was used in the infection assay. As shown in Table 1, *Wolbachia* does not inhibit viral dissemination even mosquitoes took blood with a low viral titers. The head infection rate of both HOU and HT1 strain was dropped dramatically when mosquitoes fed with dengue-infected blood at diluted viral titers. No significant difference in head infection rate was observed between HOU and HT1 in all the three diluted viral titers (P>0.05, Fisher's exact test). These results confirm that *Wolbachia* does not suppress viral dissemination in the naturally infected *Ae. albopictus*.

**Table 1:** The native *Wolbachia* does not inhibit dissemination of DENV-2 to mosquito heads in *Ae. albopictus*.

	Head infection frequency, %	
PFU/ml	Houston	HT1
10^6^	61.1 (11/18)	50.0 (9/18)
10^5^	38.9 (7/18)	38.9 (7/18)
10^4^	22.2 (4/18)	16.7 (3/18)

The HOU strain of *Ae. albopictus* and its aposymbiotic strain HT1 were infected with the blood containing DENV-2 at titers of 10^6^, 10^5^, and 10^4^ PFU/ml. At Day 14 post infection, heads of ten mosquitoes were collected and used for diagnosis of DENV-2 by RT-PCR. Data from two experiments were pooled together.

### *Wolbachia* induces expression of antimicrobial peptide DEFD in a density-dependent manner in *Ae. albopictus* cells

**Figure 5:**
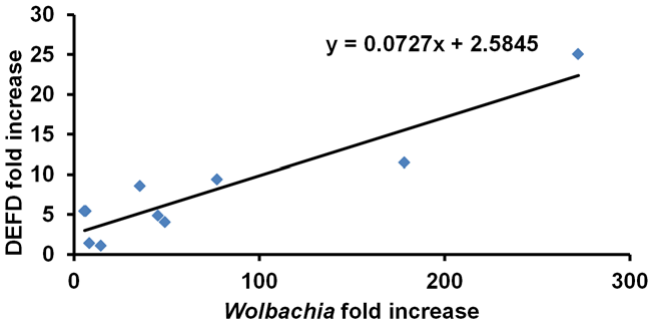
The expression of DEFD was induced by *Wolbachia* in density-dependent manner in Aa23 cell lines. Seven days after rifampicin treatment, cells were collected to measure *Wolbachia* densities and DEFD expression. Because treatment with 5 µg/ml of rifampicin for 70 h resulted in both *Wolbachia* infection and DEFD expression at lowest level, it serves as a reference for all the other treatments to calculate a fold increase in both *Wolbachia* density and DEFD expression. Each point is the mean of at least three biological replicates.

We previously show that the boosted host immunity boosted plays important roles in *Wolbachia*-mediated viral inference and antimicrobial peptide defensin is involved in this anti-dengue resistance [17]. To test whether the above *Wolbachia*-density dependent viral inhibition in Aa23 cells relates to host immunity, we measured both *Wolbachia* density and the expression of DEFD in the antibiotic treated Aa23 cells. As shown in Fig. 5, there is a positive correlation between *Wolbachia* density and the amount of DEFD transcript (r = 0.92; P = 0.0001). This supports that host immune level could contribute to *Wolbachia*-density dependent viral inhibition.

### Cells host both *Wolbachia* and DENV when *Wolbachia* density is low

**Figure 6:**
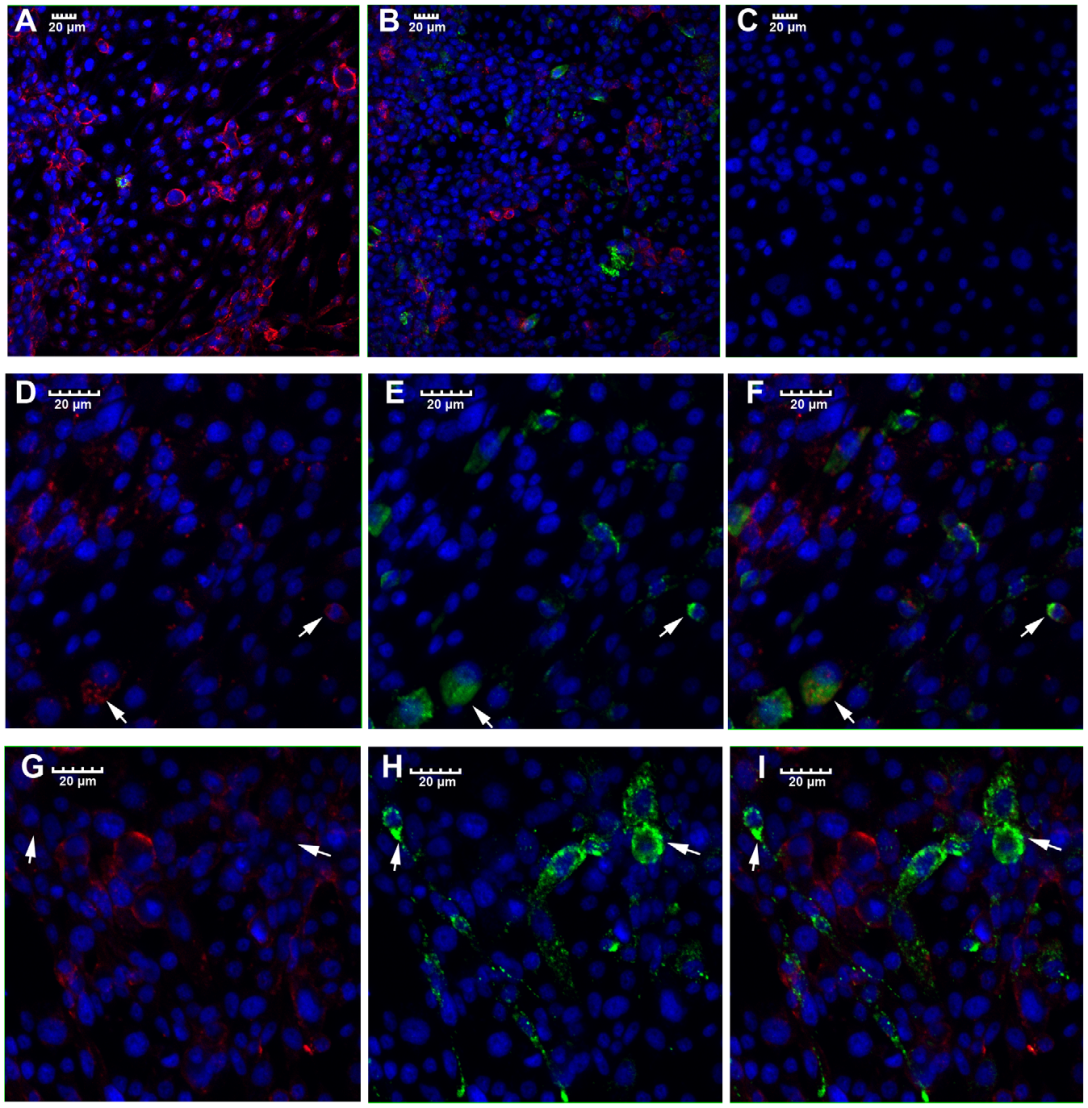
Localization of *Wolbachia* and DENV-2 in Aa23 cells. Double immunofluorescence staining of cells showing the localization of dengue virus (green) and *Wolbachia* (red). Cells were probed simultaneously with polyclonal anti-wsp antibody (*Wolbachia*) and monoclonal anti-DENV-2 antibody, followed by Alexa 594 (red) and Alexa 488 (green) conjugated antibodies, respectively. DNA (blue) is stained with DAPI. In panels (A, B, and C), the red, green and blue channels are merged. A and B show Aa23 cells with mock treatment and treatment with 5 µg/ml of rifampicin for 5 hr, respectively, followed by dengue infection. C is Aa23T cells without dengue infection (negative control). D to F or G to I is the same sample with different channel merged: D and G show only red and blue channel merged, E and H show only green and blue channel merged, F and I show all the red, green and blue merged. Aa23 cells treated with 5 µg/ml of rifampicin for 10 hr (D to F) and 40 hr (G to I), followed by dengue infection, are shown.

To study whether the above antibiotic treatment leads to a uniform change in both *Wolbachia* and DENV at the cellular level, cells at two days post-DENV infection were assayed by double immunofluorescent staining to visualize the distribution of *Wolbachia* and DENV-2 using antibodies against the wsp of *Wolbachia* and envelop protein of DENV-2. As expected, we observed *Wolbachia* was largely removed by rifampicin treatment, resulting in more cells infected by DENV-2 (Fig. 6A–C). However, such a reduction in *Wolbachia* density appears not to be even, resulting in that certain cells host much more *Wolbachia* than the others (Fig. 6A–C). Importantly, we observed that colocalization of DENV-2 and *Wolbachia* can occur in the same cells when *Wolbachia* density is low in those cells (Fig. 6D–E). For those cells that were heavily infected by DENV-2, however, they typically do not contain *Wolbachia* (Fig. 6G–I).

## Discussion

*Wolbachia* can induce a resistance to DENV and other arboviruses in *Ae. aegypti* [12], [13]. This raises a puzzle as to why mosquitoes that naturally carry *Wolbachia*, such as *Ae. albopictus*, can still serve as vectors for the arboviruses. Here, we demonstrate that *w*AlbB is able to induce resistance to DENV in *Ae. albopictus* Aa23 cell line, and such viral interference occurs in *Wolbachia* density-dependent manner. We also show that *Wolbachia* density in somatic tissues of *Ae. albopictus* is significantly lower than the transinfected *Ae. aegypti* WB1. Such a low density of *Wolbachia* is predicted not to induce viral interference in *Ae. albopictus*. Consistently, *Wolbachia* does not suppress viral dissemination in *Ae. albopictus* even when mosquitoes take an infectious blood meal at a very low viral titer. Moreover, we observed a positive correlation between *Wolbachia* density and the expression of the antimicrobial peptide DEFD. When *Wolbachia* density is low, DENV can colocalize with *Wolbachia* in the same cell hosts.

Consistent with previous studies, *Wolbachia* distributes in both somatic and germ line tissues in *Ae. albopictus* [21]. We further show that only *w*AlbB presents in midgut, salivary gland and fatbody while *w*AlbA was detected in only ovary. In this work, we focus on the above three somatic tissues because midgut and salivary gland are the two major sites for DENV to replicate and migrate through in order for mosquitoes to be infectious, and fatbody is the main immune organ to defend against foreign invaders [32]. Our results indicate that the *Wolbachia* infection in midgut and fatbody of *Ae. albopictus* may be too low to suppress viral replication in midgut and the subsequent dissemination into the other parts of mosquito body. This is supported by the lack of difference in the head infection rate between the HOU and HT1 strain. Comparison of *w*AlB infection between the dengue resistant WB1 and the susceptible HOU strain shows that there is a general reduction in *Wolbachia* density in HOU strain and the magnitude of reduction in midgut is more than the other tissues.

We observe a linear negative correlation between *Wolbachia* density and genome copy of DENV-2 in Aa23 cell line. Based on the model (y = −0.004x+3.847), *Wolbachia* density at 961.8 wsp/actin is required to completely clean DENV in Aa23 cells. With the observed *Wolbachia* copy in midgut (0.3 wsp/actin) and salivary gland (5.3 wsp/actin) of Ae. albopictus HOU strain, *Wolbachia* would have no inhibition to DENV-2 in midgut and reduced DENV-2 by 0.6% in salivary gland in this mosquito species. However, it is possible that our model will be more appropriate to be used in the *in vitro* system. *Wolbachia* strains, host cells or tissues and environment may influence the viral inhibition, resulting in a modified linear correlation. Even that, a general pattern of *Wolbachia* density dependent viral inhibition should present in different systems. When *w*MelPop-CLA was introduced into two *Ae. albopictus* cell lines, RML12 and C6/36, the cell line with a high *Wolbachia* density (C6/36) shows a strong inhibition to DENV-2 [20]. In *D. simulans*, *Wolbachia* strains that grow to high density provide the highest protection from virus infection [33]. All these indicate that *Wolbachia* density-dependent viral inhibition occurs for both native and non-native *Wolbachia* and cross different host cell types.

The linear negative correlation between *Wolbachia* density and dengue copy could lead to a simple hypothesis, in which *Wolbachia* secretes antimicrobial effector molecules into a host cell to directly inhibit pathogens. Such effectors should have a broad-spectrum activity against a variety of pathogens, including virus, plasmodium and worm. Consistently, a bacterium was recently reported to produce ROS and induce resistance to malaria parasites in *Anopheles gambiae* mosquitoes [34]. The Type IV Secretion System, which presents within *Wolbachia* [35], [36], was reported to secret different factors into host to mediate interactions between the other intracellular bacteria and their hosts [37]. Our preliminary studies show that induction of DEFD expression occurs in *Wolbachia*-density dependent manner, providing us support to further test this hypothesis.

Currently there are two major modes to explain the mechanisms of *Wolbachia*-mediated viral interference [16], [19]. The first is *Wolbachia* boosts mosquito basal immunity against viral infection, while the second is that *Wolbachia* and virus compete for the same host resource. It appears that *Wolbachia* density dependent viral inhibition could be explained by both modes. In the first mode, a high *Wolbachia* density could cause high oxidative stress in host, leading to production of more ROS for a local antiviral immune response. The low *Wolbachia* density in somatic tissues of *Ae. albopictus* is consistent with the previous observation that *Wolbachia* neither induces nor suppresses transcripts encoding antimicrobial peptides in *Ae. albopictus* [18]. In the second mode, more *Wolbachia* could lead to less host resource for virus. While it appears to be straightforward for the second mode to fit the density-dependent data, it will be challenging to identify the common host components needed by both *Wolbachia* and a variety of pathogens.

Our results indicate cell lines could be used as a simple *in vitro* system to study the mechanism of *Wolbachia*-mediated viral interference. DENV was strongly inhibited in *Wolbachia*-infected Aa23 and Aag-2 cells. Recent studies identified hundreds of host proteins that are required for DENV-2 propagation in cell lines [38]. Evidence indicates that these host proteins form dengue-associated protein interaction networks, consisting of highly interconnected proteins with closely related functions in each of replication/transcription/translation, immunity, transport and metabolism [39]. *Wolbachia* could perturb the above network to influence dengue infection. The cell culture provides us an ideal tool to study the interaction of *Wolbachia* with these dengue host proteins/networks.

In the field, a difference in *Wolbachia* density in mosquito vectors may directly influence the disease transmission. *Wolbachia* density was reported to differ largely among *Ae. albopictus* collected from the field in different locations in Thailand [40]. It is still unknown to what extent this will affect the vector status and vector competence of *Ae. albopictus*. However, conflicting results were reported on the relative susceptibility of *Ae. albopictus* versus *Ae. aegypti* to oral DENV infection [22], [41]. Some studies show that *Ae. albopictus* is more susceptible to DENV than *Ae. aegypti*, while the others show opposite results [41], [42], [43], [44], [45]. It was also reported a significant increase of susceptibility to DENV in *Ae. albopictus* with increasing generations in the laboratory [41], [45]. Although generally considered a secondary vector of dengue, *Ae. albopictus* was the primary cause of dengue epidemics in a number of areas [22], [46], [47], [48], [49]. In China, *Ae. albopictus* has long been considered the primary dengue vector. Dengue epidemics frequently occurred without *Ae. aegypti*, with 22,122 new infections, including the fatal dengue hemorrhagic fever, recorded in single year [22], [50], [51], [52]. All the above suggest vector competence and vector status of *Ae. albopictus* can vary in different locations. Future work should study the impacts of *Wolbachia* on vector status and vector competence of *Ae. albopictus*.

Our results could provide important implications for the future vector-borne disease control. First, novel control strategies can be developed to manually increase *Wolbachia* density in mosquitoes naturally carrying this bacterium. This could lead to blocking pathogen transmission to human but without a need to eradicate these mosquito species. Second, it is still unknown whether *Wolbachia* can persistently maintain infection at a level that effectively induces complete resistance to DENV in the transinfected *Ae. aegypti*. We could not exclude the possibility that adaption of a recent *Wolbachia* to a new host will finally lead to a low *Wolbachia* density, resulting in that what we see today in *Ae. albopictus* will be what happens tomorrow in those transinfected *Ae. aegypti*. Understanding how *Wolbachia* density is regulated by mosquito hosts and how the *Wolbachia* machinery controls its replication will facilitate the current effort to eliminate dengue through *Wolbachia*-based population replacement [53].
